# Estrogen inhibits colonic smooth muscle contractions by regulating BKβ1 signaling

**DOI:** 10.1371/journal.pone.0294249

**Published:** 2023-11-10

**Authors:** Jing Wen, Yu Zhao, Cheng Huang, Shengjie Li, Peidong Li, Yu Zhou, Zaihua Yan, Guangjun Zhang

**Affiliations:** 1 The Second Department of Gastrointestinal Surgery, The Affiliated Hospital of North Sichuan Medical College, Nanchong, Sichuan, China; 2 Institute of Hepatobiliary, Pancreatic and Intestinal Disease, North Sichuan Medical College, Nanchong, Sichuan, China; Indiana University School of Medicine, UNITED STATES

## Abstract

The estrogen inhibits colonic smooth muscle contractions, which may lead to constipation. However, the mechanisms of inhibition are poorly understood. Therefore, the present study examined the effect of estrogen on rat colonic smooth muscle contractions and its potential association with the large-conductance Ca^2+^-activated K^+^ channels β1 (BKβ1) subunit. Twenty-four female Sprague Dawley rats were randomly assigned to 4 groups. After 2 weeks of intervention, the contraction activity of isolated colonic smooth muscle and the expression of BKβ1 in colonic smooth muscle of rats were detected. Additionally, in order to investigate the effects of estrogen on BKβ1 expression and calcium mobilization, in vitro experiments were conducted using rat and human colonic smooth muscle cells (SMCs). BKβ1 shRNA was used to investigate whether calcium mobilization is affected by BKβ1 in colonic SMCs. To explore the relationship between ERβ and BKβ1, serial deletions, site-directed mutagenesis, a dual-luciferase reporter assay, and chromatin immunoprecipitation assays were employed. In response to E2, colonic smooth muscle strips showed a decrease in tension, while IBTX exposure transiently increased tension. Furthermore, in these muscle tissues, BKβ1 and α-SMA were found to be co-expressed. The E2 group showed significantly higher BKβ1 expression. In cultured colonic SMCs, the expression of BKβ1 was found to increase in the presence of E2 or DPN. E2 treatment reduced Ca^2+^ concentrations, while BKβ1 shRNA treatment increased Ca^2+^ concentrations relative to the control. ERβ-initiated BKβ1 expression appears to occur via binding to the BKβ1 promoter. These results indicated that E2 may upregulate BKβ1 expression via ERβ and inhibit colonic smooth muscle contraction through ERβ by directly targeting BKβ1.

## Introduction

Chronic constipation (CC) is a serious gastrointestinal dysfunction caused by many factors, such as dietary and lifestyle factors or colon propulsion or rectal emptying disorders [1]. CC affects 15% of adults worldwide, with a female-to-male ratio of 2:1 [2, 3]. Furthermore, studies have shown that estrogen (E2) can inhibit gastrointestinal emptying and colonic smooth muscle contractions [4, 5]. This can possibly explain why the female population is more likely to suffer from constipation than males. However, its specific mechanism of action has not yet been fully elucidated.

E2 has been implicated in multiple biological functions, with its actions mediated through nuclear estrogen receptor α (ERα) and β (ERβ), or by membrane proteins, including a hypothetical membrane estrogen receptor [6, 7]. Interestingly, ERβ is the primary estrogen receptor found in the gut [8, 9] and plays a major role in mediating colonic motor function [10, 11]. However, it is not yet known what the exact function of ERβ is in the colon.

Smooth muscle contraction is closely related to excitation-contraction coupling, with the large conductance calcium-activated potassium (BK) channel playing a crucial role in the process of depolarization and repolarization [12, 13]. In smooth muscle tissue, the BK channel is composed of an α-subunit and a smooth muscle-specific β1-subunit [14, 15]. BKβ1 is a key factor in BK channel electrophysiology that increases the sensitivity of BKα to Ca^2+^ and voltage and subtly activates the BK channel within the physiological levels of the membrane potentials and intracellular calcium ([Ca^2+^]i) concentration [16–19]. Furthermore, recent studies have shown that E2 acutely modulates the function of BK channels in coronary artery [20, 21]. To the best of our knowledge, it has not been confirmed whether estrogen regulates BKβ1 in the colon.

In the present study, estrogen receptor 2 (ESR2), which encodes ERβ, was predicted to act as a transcription factor that can interact with the potassium calcium-activated channel subfamily M regulatory beta subunit 1 (KCNMB1; encodes BKβ1) promoter using bioinformatics tools. In a previous study, a defect in the function of the BKβ1 subunit resulted in distal colonic smooth muscle impaired colonic motility and caused constipation [22]. Therefore, we concluded that the ERβ/BKβ1 signaling pathway may play an important role in the regulation of colonic motility. Overall, this study was designed to examine the role of ERβ/BKβ1 signaling in E2-induced colonic contractile activity inhibition and the possible mechanisms behind this effect.

## Material and methods

### Experimental groups and interventions

Rats used in this study were handled in strict accordance with the National Institutions of Health Guide for the Care and Use of Laboratory Animals. Rats were obtained from the Experimental Animal Center, North Sichuan Medical College (Nanchong, China). Animals were housed in cages under standard conditions with a 12-hour light/dark cycle (7:00am-7:00pm), room temperature, and 40% relative humidity. A total of 24 female Sprague-Dawley rats (180–200 g) were randomized into four groups as follows: C group (control), E2 group, E2+EI (estrogen inhibitor) group, and bovine serum albumin-conjugated estrogen (BSA-E2) group (n = 6 per group). All rats were bilaterally ovariectomized (OVX) after anesthesia. Following bilateral ovariectomies, all rats were implanted with silicone tubes (30-mm length, 2-mm inner diameter, and 4-mm outer diameter) and the orifices were sealed with Type A adhesive (Dow Corning, Midland, MI). In the different groups, different solutions were administered for 2 weeks via the tubes and included corn oil for C group; 0.3 g/l estrogen (ab120657; Abcam, Cambridge, UK) for the E2 group for maintaining serum levels of estrogen in the physiological range [23, 24]; 0.3 g/l each of estrogen and an estrogen receptor inhibitor (ICI182780, SantaCruz, CA, USA) for the E2+EI group; and 0.3 g/l of BSA-E2 (Sigma-Aldrich, St. Louis, MO) in the BSA-E2 group. Animal experimentation was reviewed and approved by the North Sichuan Medical College Animal Experimentation Ethics Committee (approval no. 202206).

### Cell isolation, culture, and treatment

Rat colons were dissected and repeatedly washed with HEPES Ringer solution (PHR1428; Sigma-Aldrich). The samples were then transferred to centrifuge tubes containing 0.1% type II collagenase (1148090; Sigma-Aldrich) and 0.01% soybean trypsin inhibitor (T6522; Sigma-Aldrich) in Dulbecco’s Modified Eagle’s medium (Invitrogen). Centrifugation was performed at 37°C for 20 min, followed by a 5-min centrifugation. Aliquots of cells were resuspended in DMEM containing 10% fetal calf serum (Sigma-Aldrich), penicillin (100 U/ml; Invitrogen) and streptomycin (100 μg/ml; Invitrogen) and stored for cell culture at 37°C. For the experiments, early passage (P2–P3) cells that had reached about 80% confluency were incubated in DMEM with 1% FBS for 24 h, followed by treatment with different E2 concentrations for various lengths of time. The colonic SMCs were treated with DMSO, E2, ICI 182780+E2, PPT (ERα-selective agonist), DPN (ERβ-selective agonist), or BSA-E2, with BKβ1 measured following treatments. Human colonic SMCs were purchased from Procell Life Science & Technology Co., Ltd. (Wuhan, China) and cultured with Smooth Muscle Cell Medium (ScienCell Research Laboratories, Carlsbad, CA, USA). The treatment methods for the human colonic SMCs were the same as described for the rat colonic SMCs.

### Measurement of the contraction of colonic smooth muscles

After the silicone tubes were implanted for 14 days, all of the rats were euthanized by cervical dislocation, and the distal colonic muscle strip tissues were harvested. Tissues from the distal colon were dissected free and cut transversely into 2 cm x0.3cm muscle strips. Each strip was placed in an organ bath containing Tyrode’s solution and maintained continuously at 37°C and aerated with 95% oxygen and 5% carbon dioxide. Colon muscle specimens were ligatured at both ends with a medical thread. A tonotransducer connected one end of the specimen to a physiological recorder and the other end to the bath bottom. A BL-420F Physiological Signal Collection and Handling System (Chengdu, China) was used to record the spontaneous contractions in each strip following stimulation with 1 g of pre-tension. Upon reaching the maximum contraction, the muscle strips were measured for tension. Strips were snap frozen in liquid nitrogen and stored at −80°C until further use.

### Experimental protocol

To stimulate the tissue, 1 x 10−4 mol/l acetylcholine chloride (Ach; Sigma-Aldrich) was added after system stability had been achieved for 1 h. Those tissues that failed to respond to Ach treatment were discarded. Once Ach produced a response, immediately measure the contraction activity of colonic muscle strips in each group for 10 min. In another experiment, before exposure to E2(1 μmol/l), circular muscle strips were preincubated for an additional 6 min with IBTX (0.1 μmol/l).

### Immunofluorescence

Frozen strips were rinsed with PBS and fixed with 100% cold acetone at −4°C. The frozen samples were blocked with BSA for 1 h and incubated with primary antibodies against BKβ1 (rabbit, diluted 1:100; ab3587, Abcam) and α-smooth muscle actin (α-SMA; mouse, diluted 1:100; Abcam), with anti-α-SMA used to identify smooth muscles. After removing the primary antibodies, the samples were then rinsed three times in PBS (5 min per wash). Samples were then incubated with secondary antibody, goat Cy3-labeled anti-rabbit IgG (diluted 1:100; Beyotime, Shanghai, China) and goat FITC-labeled anti-mouse IgG (diluted 1:100; Beyotime) and allowed to incubate at 37°C in the dark for 1 h. Next, the samples were rinsed three times with PBS (5 min per wash) and stained with 4’,6-diamidino-2-phenylindole (DAPI; C1002, Beyotime) for nuclear visualization. Finally, the samples were mounted on a glass slide after being quenched with an anti-fluorescence quencher and observed under a fluorescence microscope.

### Ca^2+^ fluorescence intensity

To determine changes in intracellular Ca^2+^ ([Ca^2+^]i) within the smooth muscle cells (SMCs), samples were incubated with the fluorescent probe Fluo-3/AM (F23915; Invitrogen, USA) dissolved in dimethyl sulfoxide (DMSO), with the probe stored at −20°C in the dark. SMCs were cultured in laser confocal dishes, washed with PBS and incubated with the probe for 40 min in the dark. Samples were rinsed following the incubation and a confocal microscope was used to scan the dishes every 20 s. The variation in [Ca^2+^]i fluorescence intensity was expressed as F/F0, where F0 represents the intensity of the first stable image.

### Quantitative real-time PCR (qPCR)

Total RNA was extracted from cultured SMCs using a TRIzol kit (Invitrogen) and cDNA was synthesized using a reverse transcription kit (Invitrogen) according to the manufacturers’ instructions. Next, qPCR was performed using SYBR Green PCR Master Mix (Takara Bio Group, Japan) according to the manufacturer’s protocols. Samples were analyzed using a Roche LightCycler 480 II Real-Time PCR System (Roche, IN, USA) under the following thermocycling conditions: 95˚C for 10 min; followed by 40 cycles of 95˚C for 30 s, 60˚C for 30 s and 72˚C for 30 s; and a final extension at 72˚C for 2 min. The 2^-∆∆Cq^ method was used to measure target genes expression [25]. Results were normalized to GAPDH and forward and reverse primer sequences are listed in Table 1.

**Table 1:** Primer and shRNA sequences used in the present study.

Primer/shRNA name	Primer sequence	Enzyme
Primers for qRT-PCR		
Human:		
BKβ1 sense	5’-CTTCTCCGCACCTCGGGGGA-3’	
BKβ1 antisense	5’-CGGTCAGCAGGAAGGTGGGC-3’	
Rat:		
BKβ1 sense	5′-ACCCATGCCTTTGGGTCAAT-3′	
BKβ1 antisense	5′-ATAGAGGCGCTGGTACACAA-3′	
GAPDH sense	5′- GCGAAAGCATTTGCCAAGAA-3′	
GAPDH antisense	5′-GGCATCGTTTATGGTCGGAAC-3′	
shRNA sequences		
Human:		
shBKβ1 sense	5’-ccgg**CCACCTGATTGAGACCAACAT**CTCG AGATGTTGGTCTCAATCAGGTGGTTTTTg-3’	
shBKβ1 antisense	5’-aattcAAAAACCACCTGATTGAGACCAACAT CTCGAGATGTTGGTCTCAATCAGGTGG-3’	
Rat:		
shBKβ1 sense	5’-ccgg**GGACAACTACCAGACAGCCTT**CTCG AGAAGGCTGTCTGGTAGTTGTCCTTTTTg-3’	
shBKβ1 antisense	5’-aattcAAAAAGGACAACTACCAGACAGCCTT CTCGAGAAGGCTGTCTGGTAGTTGTCC-3’	
Primers for KCNMB1 promoter construct		
(-1,783/+98)KCNMB1 sense	5’-TATAGGTACCatccttggaaaattgctctt-3’	KpnI
(-647/+98)KCNMB1 sense	5’-TATAGGTACCcttcagctcagttggtcc -3’	KpnI
(-296/+98)KCNMB1 sense	5’-TATAGGTACCtttctggcatctttgccag-3’	KpnI
Antisense	5’-ATATAAGCTTtctcccatgtgccgtcctcc-3’	HindIII
Primers for KCNMB1 promoter site-directed mutagenesis		
Binding site 1 mutation sense	5’-TGAGAGTGctgtctttcaattTGAGTGGC-3’	
Binding site 1 mutation antisense	5’-GCCACTCAaattgaaagacagCACTCTCA-3’	
Binding site 2 mutation sense	5’-TTGCTCAGttctgtgacaagtGTAAGTGC-3’	
Binding site 2 mutation antisense	5’-GCACTTACacttgtcacagaaCTGAGCAA-3’	
Primers used for ChIP in the KCNMB1 promoter		
Distant region sense	5’- ATTCAGAGTATCACGAGCAA-3’	
Distant region antisense	5’-CCACTCAGAAGCAGGAAA-3’	
Binding site 1 sense	5’-GAAAGGGGTGGAGAAGGGC-3’	
Binding site 1 antisense	5’-CGAGCTCACCACCTTCCTC-3’	
Binding site 2 sense	5’-GAGGTGCCATAACTTGCT-3’	
Binding site 2 antisense	5’-CCCTGGCATATCCACGCT-3’	

Text in bold indicates the core sequence. ChIP, chromatin immunoprecipitation; sh/shRNA, short hairpin RNA

### Western blot analysis

Proteins lysates were obtained for the cultured SMCs and the colonic smooth muscle tissues using RIPA lysis buffer (Beyotime), with protein concentrations assessed using a BCA kit (Beyotime Institute of Biotechnology). Proteins (40 μg/lane) were separated on a 10% agarose gel via SDS-PAGE and then transferred to a PVDF membrane. The membranes were blocked with 5% skim milk for 1.5 h at room temperature, and then incubated overnight at 4˚C with primary anti-BKβ1 antibody (1:100; ab3587, Abcam). Next, the membranes were incubated for 1 h at 37˚C with horseradish peroxidase–conjugated secondary antibodies. Electrochemiluminescence (ECL) reagent (WBKLS0500; Millipore Sigma) was used to visualize the protein bands. GAPDH was used as the internal reference and all samples were evaluated in triplicate.

### Establishment of lentiviruses and plasmid construction

Lentiviral vectors with a human ESR2 sequence were established using a pLVX-EGFP-IRES-Puro plasmid (cat. no. BR684; Hunan Fenghui Biotechnology Co., Ltd.) and were designated as LV-ESR2. An empty vector control was also employed and designates as LV-control. A standard procedure was followed for the establishment of the plasmid vectors, with the utilized primers listed in Table 1. PCR was used to amplify the KCNMB1 promoter sequence (−1,783/+98) from human colonic SMC genomic DNA using a genomic DNA extraction kit (Invitrogen). In vector construction, forward and reverse primers were integrated at the 5’- and 3’-ends of the KpnI and Hind III sites, respectively. PCR products were inserted between the digested Hind III and KpnI sites of the pGL3-Basic vector (Promega Corporation). Moreover, 5’-flanking KCNMB1 promoter deletion mutants [(−1,783/+98) KCNMB1; (−647/+98) KCNMB1; and (−296/+98) KCNMB1] were established using the (−1,783/+98) KCNMB1 vector as the template. A QuikChange II Site-Directed Mutagenesis kit (Stratagene; Agilent Technologies, Inc.) was used to make mutations in the ESR2-binding site within the KCNMB1 promoter. To verify successful vector construction, DNA sequencing was performed by Sangon Biotech Co., Ltd. (Shanghai, China).

### Chromatin immunoprecipitation (ChIP) assay

In order to verify the binding of ERβ to the primer regions of KCNMB1, a ChIP assay kit (ab500; Abcam) was used according to manufacturer’s protocols. Before the ChIP experiment, human colonic SMCs were treated with 50 nmol/l E2. To extract chromatin from human colonic SMCs, sonication was used, followed by immunoprecipitation using 1 μg of anti-ERβ (ab3576, Abcam) or normal goat IgG. The purified DNA obtained from the immunoprecipitation or the original chromatin was then amplified using PCR, with all experiments performed in triplicate. Sites 1 and 2 are two potential ESR2-binding sites. The sequence region of site 1: (-1783/-647) KCNMB1. The sequence region of site 2: (-647/-296) KCNMB1. The primer sequences used in ChIP were shown in Table 1.

### Transient transfection and luciferase assay

ESR2 expression plasmids were constructed by cloning ESR2 DNA into a pCMV-tag2A vector (Agilent Technologies, Inc.). Human colonic SMCs were co-transfected with an expression plasmid, control (pCMV-Tag) or pCMV-ESR2, and a reporter plasmid (Promega Corporation). At 5 h post transfection, human colonic SMCs were washed and then incubated in medium containing 1% FBS for 48 h to recover. In addition, human colonic SMCs were treatment with 50 nmol/l E2. After serum-starvation, the human colonic SMCs were analyzed using a Dual-Luciferase Assay Kit (Beyotime Biotechnology) according to the manufacturer’s instructions. Briefly, the transfected cells were lysed and centrifuged at 72000 × g for 120 min at 4˚C in Eppendorf microcentrifuge tubes. The relative luciferase activities were determined using a TD20/20 luminometer (Turner Diagnostics, Sunnyvale, CA, USA). Luciferase activity was normalized to Renilla luciferase activity, with three biological replicates examined for each sample.

### Stable knockdown of BKβ1 in colonic SMCs

Lentiviral packaging was performed by transfecting HEK293T cells with pLKO.1-based vectors encoding short hairpin RNA (shRNA) specific to the BKβ1 gene (Table 1). Lipofectamine 3000 Regent (Invitrogen) was used to package envelope plasmids pMD2.G and psPAX2 into lentiviruses. For transduction, viral supernatants were added to the colonic SMCs, followed by the addition of 8 μg/mL polybrene, a 24-h incubation with puromycin (1 μg/mL), and a final 72-h period or selection. PCR was used to confirm the knockdown.

### Bioinformatics analysis

The University of California Santa Cruz (UCSC) Genome Browser (http://genome.ucsc.edu) was utilized to find the KCNMB1 promoter sequence. The JASPAR website (https://jaspar.genereg.net/) was used to analyze the KCNMB1 promoter and predict ESR2 binding sites.

### Statistical analysis

This study used SPSS version 23.0 (IBM, Chicago, IL, USA) for all statistical analyses. In all cases, values are shown as mean ± standard error of the mean (SEM), unless otherwise stated. When comparing two groups, a paired or unpaired Student’s *t*-test was employed, and a one-way ANOVA with Dunnett’s post hoc test or Tukey’s post hoc test was used to compare multiple groups. GraphPad Prism (version, 9; GraphPad Software, Inc.) was used to produce graphs. Statistical significance was defined as *P* < 0.05, with experiments repeated in triplicate to ensure accuracy.

## Results

### Contractile activity of colonic smooth muscle

**Fig 1:**
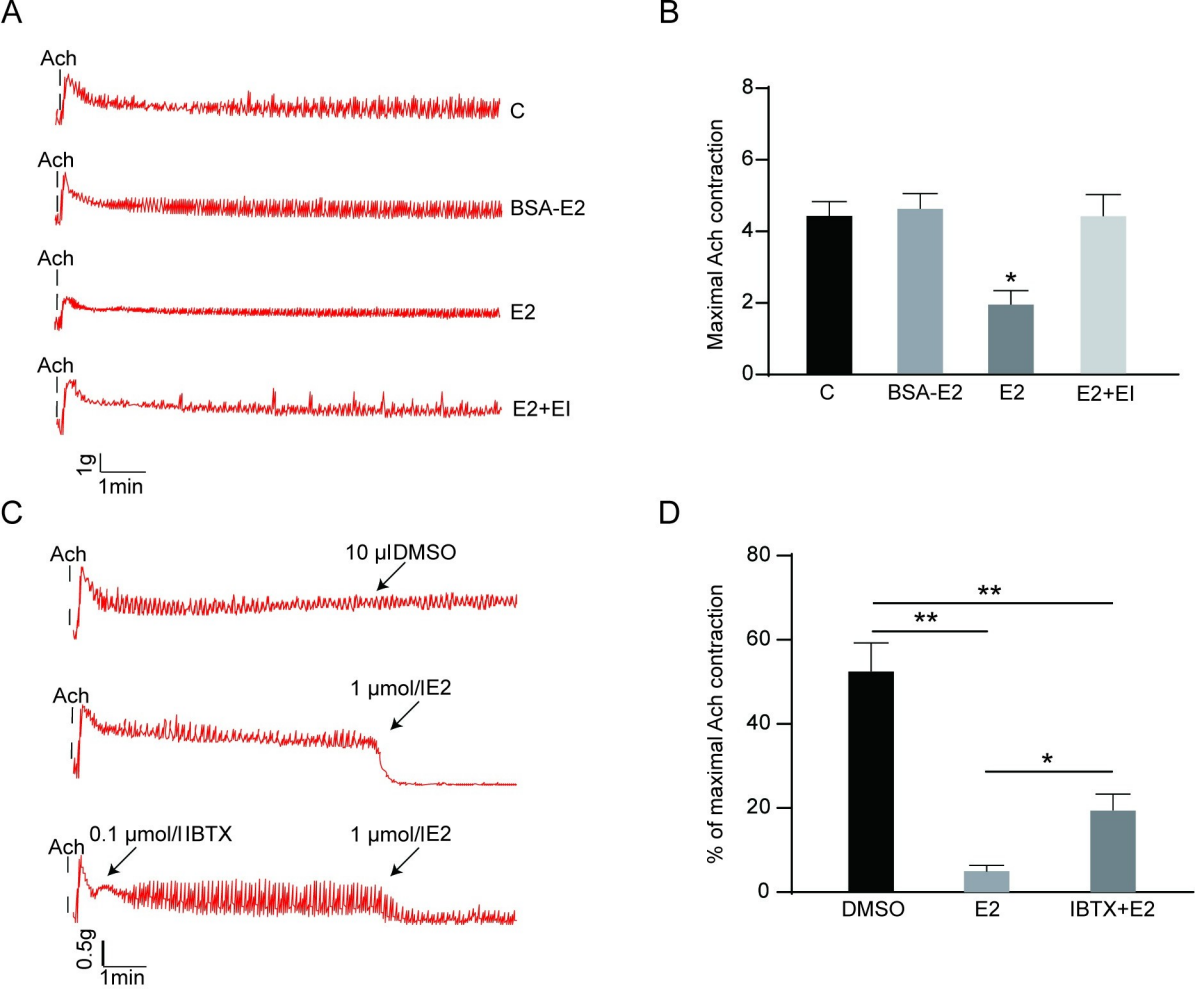
Iberiotoxin-sensitive BKβ1 is involved in E2-induced colonic SMC relaxation. **A** Contractile activity of colonic SMC in each group (n = 6 each). **B** Detection of the maximal contraction after Ach stimulation. *P < 0.05 when compared with C, BSA-E2 and E2+EI groups. **C** Representative trace showing that E2 (1 μmol/l) inhibits rat colonic SMC contractions. Preincubation with Iberiotoxin (IBTX, 1 μmol/l), a BKβ1 blocker, partially reversed the contractile E2-induced inhibition (n = 6 each). **D** Results are presented as a percentage, which is expressed as the ratio of the contraction amplitude of smooth muscles after DMSO or estrogen treatment to the maximum value of ACH-induced contraction. All data are displayed as mean ± SEM. *P < 0.05 and **P < 0.01.

To probe the relationship between E2 and colonic contraction, *in vitro* organ bath-based studies were performed. After exposure to Ach (1 x 10−4 mol/l), measure the contraction activity of colonic muscle strips in each group for 10 min. Our data showed that colonic smooth muscle contractile activity was significantly lower in the E2 group relative to the other groups. However, no significant differences were observed between the BSA-E2, control and E2+EI groups (Fig 1A and 1B). To determine whether E2-induced relaxation of colonic smooth muscle is related to BKβ1 activation, the BKβ1 inhibitor iberiotoxin (IBTX, 0.1 μmol/l) was utilized. The results showed that IBTX partially reverses E2-induced relaxation, thus suggesting that the BKβ1 subunit may be a potential estrogen target (Fig 1C and 1D).

### BKβ1 distribution and expression in isolated rat colonic smooth muscles stripes

**Fig 2:**
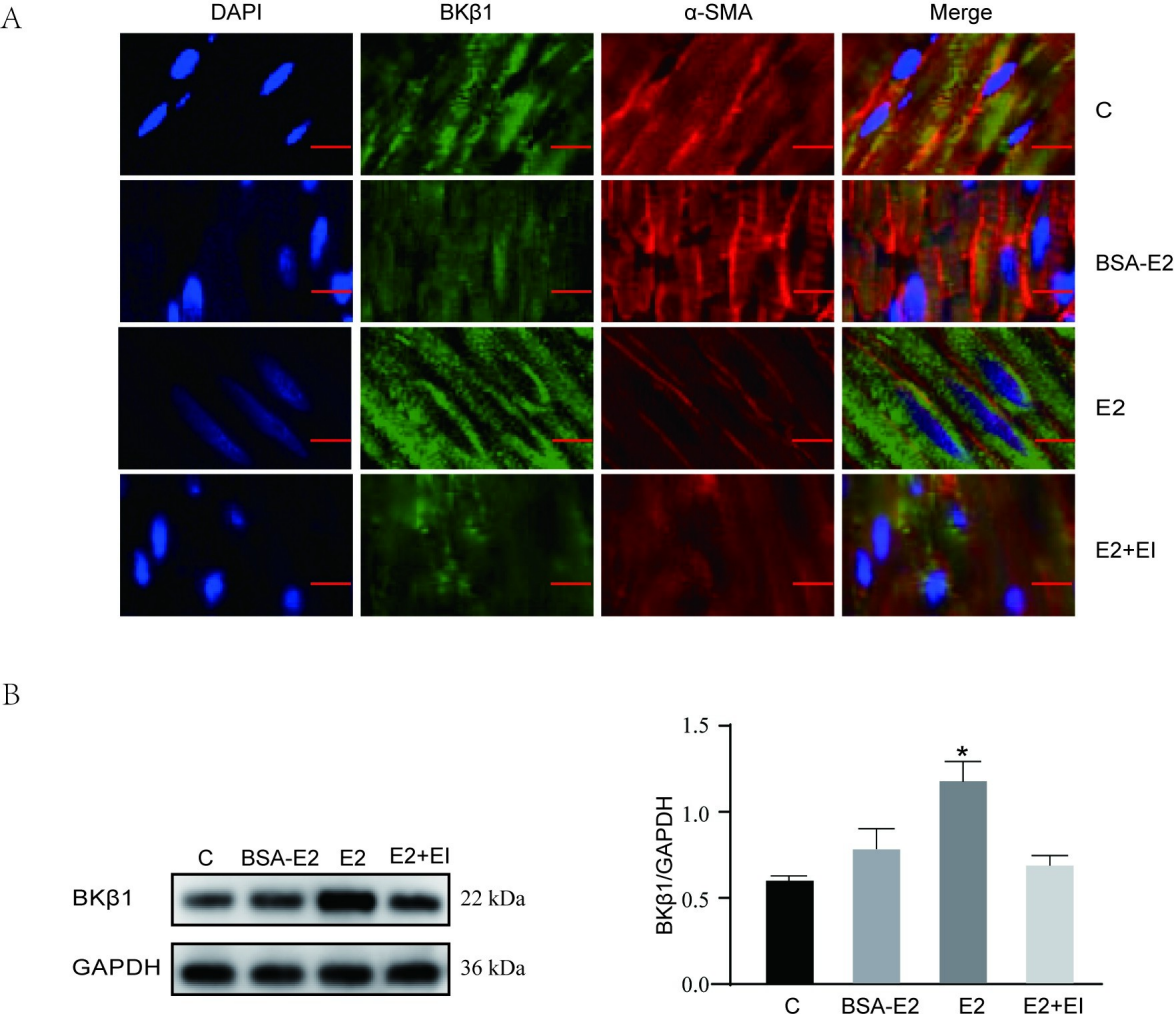
BKβ1 expression and distribution in rat colonic smooth muscle. **A** Representative double immunofluorescence image showing BKβ1 expression (green) and α-SMA (red) in four groups (n = 6 each) of colonic smooth muscle (immunofluorescence double labeling, ×200). Scale bar = 10 μm. **B** Left panel: BKβ1 protein expression as showed by western blotting in four groups; right panel: quantification of BKβ1 protein levels normalized to GAPDH, n = 6. All data are displayed as mean ± SEM. *P < 0.05 when compared with C, BSA-E2 and EI groups.

Previous studies have shown that BKβ1 widely distributed in smooth muscle tissue. Hence, immunofluorescence and western blot analyses were used to examine BKβ1 distribution and expression in rat distal colonic smooth tissue. The results showed that BKβ1 and α-SMA are co-expressed in colonic smooth muscle tissue. Moreover, in the E2 group, the fluorescent intensity for BKβ1 was higher than the other groups, with no significant differences in BKβ1 fluorescence intensity observed among the E2+EI, BSA-E2 and control groups (Fig 2A and S1 Fig). These findings were further confirmed via western blot analysis (Fig 2B). S1 Fig provides the quantified fluorescence intensity values for each group of BKβ1.

### Optimization of E2 administered concentration and incubation time

**Fig 3:**
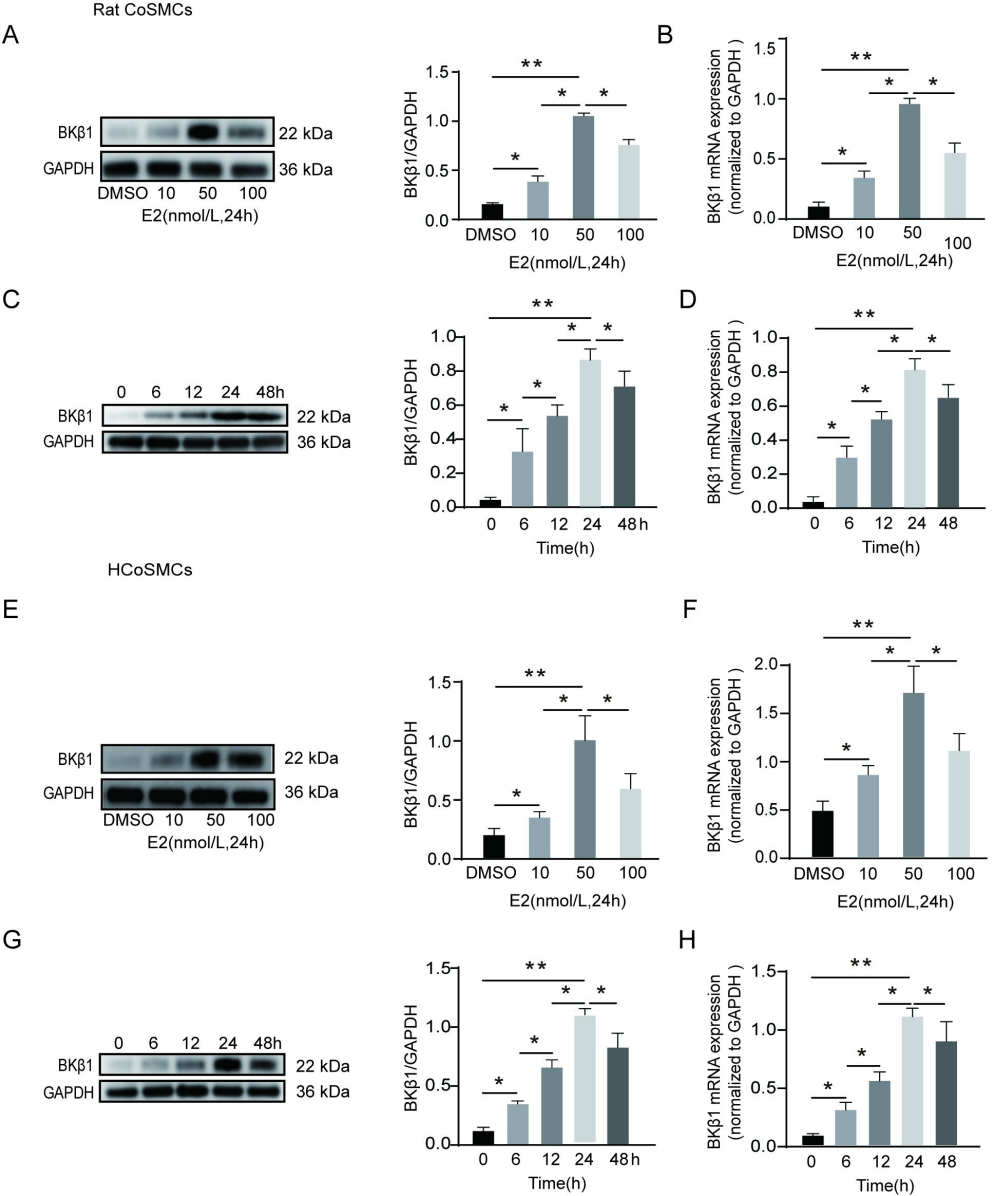
E2 treatment increases BKβ1 expression in colonic SMCs in a concentration- and time-dependent manner. **A** E2 was administered to rat CoSMCs for 24 h at 0, 10, 50, or 100 nmol/l. Left panel: representative protein bands; right panel: quantitative analysis for BKβ1; data were normalized to GAPDH (n = 3). **B** BKβ1 mRNA expressional analysis using qPCR in rat CoSMCs. Data are expressed relative to GAPDH mRNA transcript levels (n = 3). **C** Rat CoSMCs were treated with 50 nmol/l of E2 for 0, 6, 12, 24 or 48 h. Left panel: representative protein bands; right panel: quantitative analysis for BKβ1 (n = 3). **D** Total BKβ1 mRNA as detected by qPCR in rat CoSMCs (n = 3). **E** E2 was administered to HCoSMCs for 24 h at 0, 10, 50, or 100 nmol/l. Left panel: representative protein bands; right panel: quantitative analysis for BKβ1 (n = 3). **F** BKβ1 mRNA expression in HCoSMCs analyzed using qPCR (n = 3). **G** HCoSMCs treated with 50 nmol/l of E2 for 0, 6, 12, 24 or 48 h. Left panel: representative protein bands; right panel: quantitative analysis for BKβ1 (n = 3). **H** Total BKβ1 mRNA expression in HCoSMCs as detected by qPCR (n = 3). Mean ± SEM. *P < 0.05 and **P <0.01. Rat CoSMCs: rat colonic smooth muscle cells; HCoSMCs: human colonic smooth muscle cells.

In Fig 2, we found that E2 can upregulate the expression of BKβ1. To screen out the best experimental concentration and time of E2, western blot and qPCR analyses were performed in colonic SMCs. We firstly screened the best concentration of E2. Rat colonic SMCs were treated with 0–100 nmol/l E2, and an E2 concentration of 50 nmo/l resulted in the highest BKβ1 protein and mRNA expression (Fig 3A and 3B). Next, rat colonic SMCs were incubated with 50 nmol/l of E2 for 0–48 h. Cells that were incubated from 0–24 h showed a time-dependent increase in BKβ1 protein and mRNA expression, with the highest level achieved at 24 h. However, expression levels began to decrease when approaching 48 h of incubation (Fig 3C and 3D). Thus, an E2 concentration of 50 nmol/l and a 24-h incubation were deemed optimal, with similar results obtained in human colonic SMCs under the same conditions (Fig 3E–3H). Based on our concentration and time screening results, we selected the optimal concentration of 50 nmol/l and the optimal time of 24 h for further experiments.

### Role of ERβ in E2-induced BKβ1 expression

**Fig 4:**
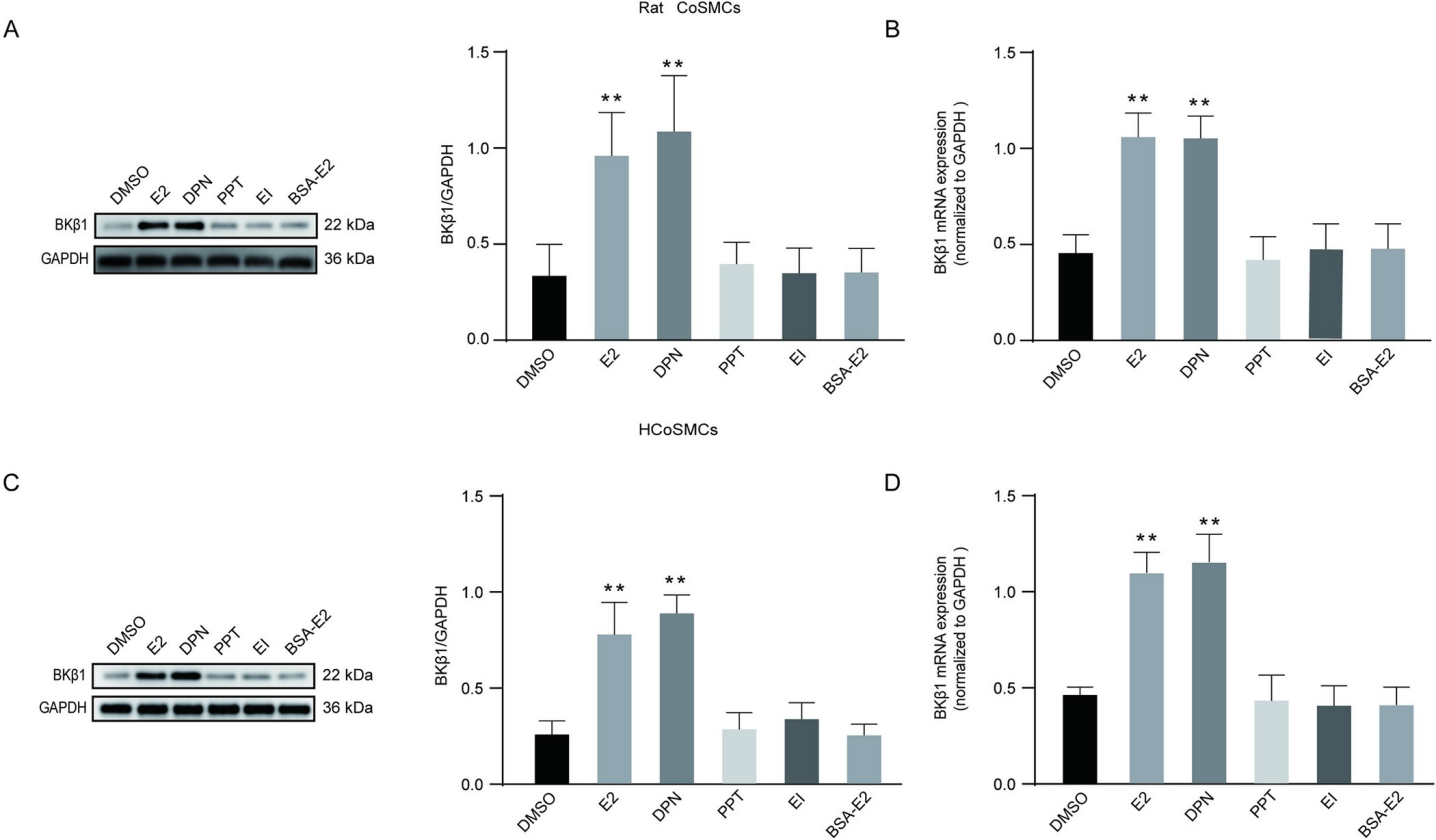
E2 promoted BKβ1 expression in SMCs in an ERβ-dependent manner. SMCs were incubated with DMSO (2 μl), E2 (50 nmol/l), PPT (1 μmol/l), DPN (1 μmol/l), ICI 182780 (1 μmol/l) + E2 (50 nmol/l), or BSA-E2 (50 nmol/l) for 24 h. **A** Western blot analysis of BKβ1in rat CoSMCs. Left panel: representative protein bands; right panel: quantitative analysis for BKβ1 (n = 3). data were normalized to GAPDH. **B** BKβ1 mRNA expressional analysis using qPCR in rat CoSMCs. Data are expressed relative to GAPDH mRNA transcript levels (n = 3). **C** Western blot analysis of BKβ1in HCoSMCs. Left panel: representative protein bands; right panel: quantitative analysis for BKβ1 (n = 3). BKβ1 expression was normalized to GAPDH (loading control). **D** BKβ1 mRNA expression in HCoSMCs analyzed using qPCR. Data are expressed relative to GAPDH mRNA transcript levels (n = 3). All data are displayed as mean ± SEM. **P < 0.01 compared with DMSO, PPT, ICI+E2 and BSA-E2.

The ER family includes plasma membrane-associated ERs and nuclear ERs (nERs), with ERα and ERβ being two subtypes of nERs. To examine which subtype is responsible for BKβ1 protein expression in the presence of E2, rat and human colonic SMCs were divided into six groups and administered different treatments. In rat colonic SMCs, treatment with DPN (ERβ-selective agonist) markedly increased BKβ1 protein and m RNA expression (Fig 4A and 4B), with a result that was similar to E2 treatment noted. However, a significant statistical difference did not exist between the other groups. In human colonic SMCs, the expression levels of BKβ1 protein and mRNA were significantly increased after incubation with DPN and E2, and there was no significant difference between the other groups (Fig 4C and 4D). Collectively, these data suggest that E2 stimulates BKβ1 protein expression via ERβ in rat and human colonic SMCs.

### The effect of E2 treatment on [Ca^2+^]i concentration

**Fig 5:**
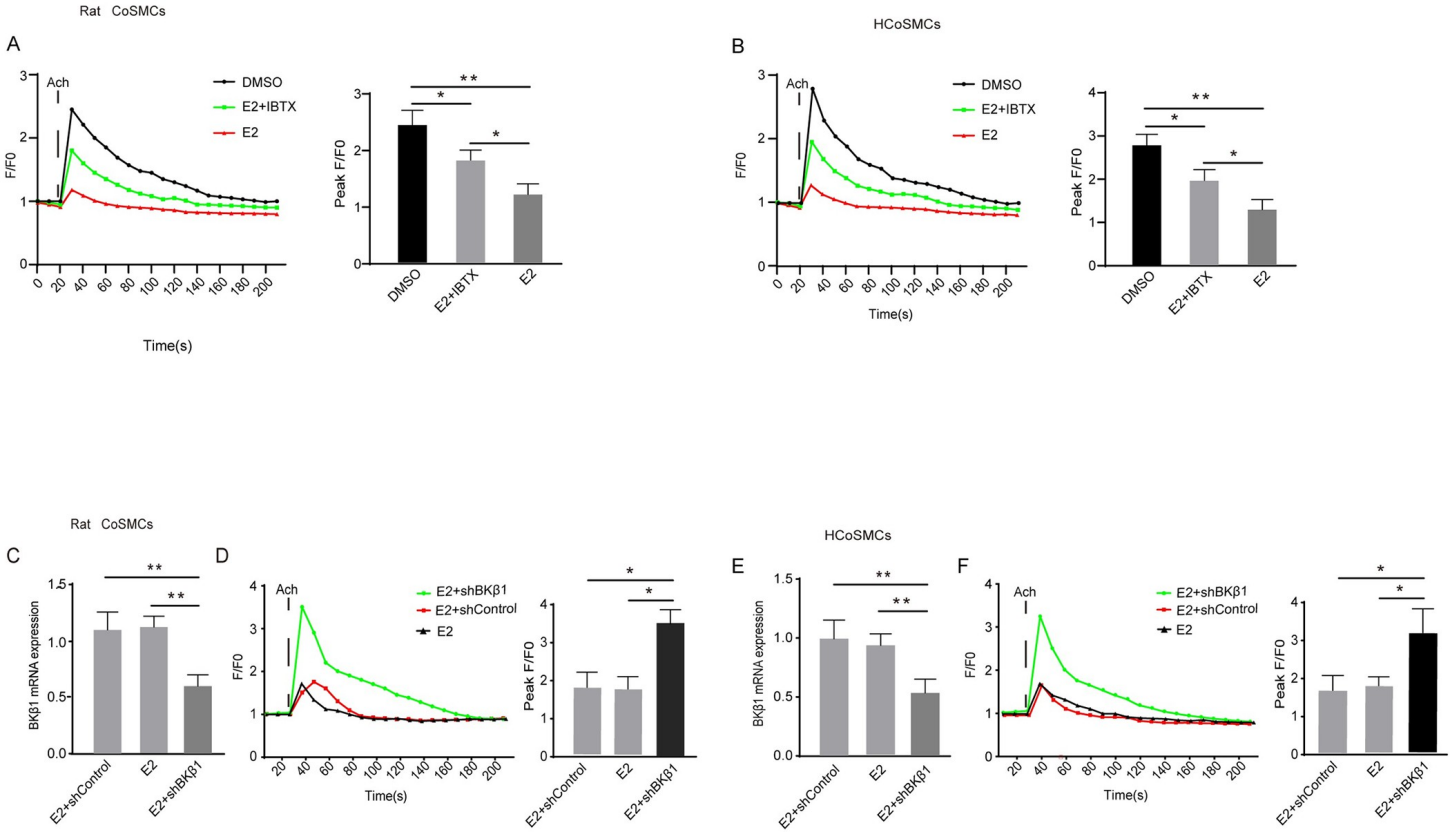
Evaluation of the effect of E2 on [Ca^2+^]i mobilization in SMCs after Ach stimulation. **A** Changes in fluorescence intensity due to [Ca^2+^]i relative to the baseline (F/F0) in rat CoSMCs treated with DMSO, E2 + IBTX, or E2. Left panel: fluorescence intensity of [Ca^2+^]i; right panel: quantitative analysis of peak F/F0 and representative image of fluorescence at peak F/F0 from three independent experiments (n = 3). **B** Changes in fluorescence intensity due to [Ca^2+^]i relative to the baseline (F/F0) in HCoSMCs treated with DMSO, E2 + IBTX, or E2. Left panel: fluorescence intensity of [Ca^2+^]i; right panel: quantitative analysis of peak F/F0 and representative image of fluorescence at peak F/F0 from three independent experiments (n = 3). F0 was derived from the average intensity of the first 0–20 s. **C** BKβ1 mRNA levels in rat CoSMCs after transfection with BKβ1 shRNA for 48 h (n = 3). **D** Changes in fluorescence intensity due to [Ca^2+^]i relative to the baseline (F/F0) in rat CoSMCs treated with BK β1-subunit knockdown. Left panel: fluorescence intensity of [Ca^2+^]i; right panel: quantitative analysis of peak F/F0. F0 was derived from the average intensity of the first 0–30 s. **E** BKβ1 mRNA levels in HCoSMCs after transfection with BKβ1 shRNA for 48 h (n = 3). **F** Changes in fluorescence intensity due to [Ca^2+^]i relative to the baseline (F/F0) in HCoSMCs treated with BK β1-subunit knockdown. Left panel: fluorescence intensity of [Ca^2+^]i; right panel: quantitative analysis of peak F/F0. All data are displayed as mean ± SEM. *P < 0.05 and **P < 0.01.

To investigate whether E2 affects Ca^2+^ mobilization, SMCs were incubated *in vitro* with a DMSO control or E2. Upon exposure to Ach, cytosolic [Ca^2+^]i increased rapidly and transiently, with levels peaking at 30 s and then gradually returning to baseline after 3 min. When compared to the control groups, E2 markedly decreased the Ach-induced rise in [Ca^2+^]i. Furthermore, pre-treatment with IBTX partially reversed the E2-induced reduction in Ca^2+^ influx (Fig 5A and 5B). These findings suggest that E2 may inhibit [Ca^2+^]i concentration via BKβ1.To further confirm whether E2 induces [Ca^2+^]i mobilization through BKβ1, a rescue experiment was performed using BKβ1 shRNA in SMCs. Transfection with the BKβ1 shRNA markedly reduced BKβ1 mRNA expression (Fig 5C and 5E) when compared with E2 group and E2+shControl group as expected. When examining [Ca^2+^]i concentrations in the SMC shRNA knockdown model, a rapid and significant increase in [Ca^2+^]i was observed relative to the E2 group and E2+shControl group (Fig 5D and 5F). Based on these findings, the Ca^2+^ signals in SMCs appear to be negatively regulated by BKβ1.

### KCNMB1 is a direct transcriptional target of ESR2

**Fig 6:**
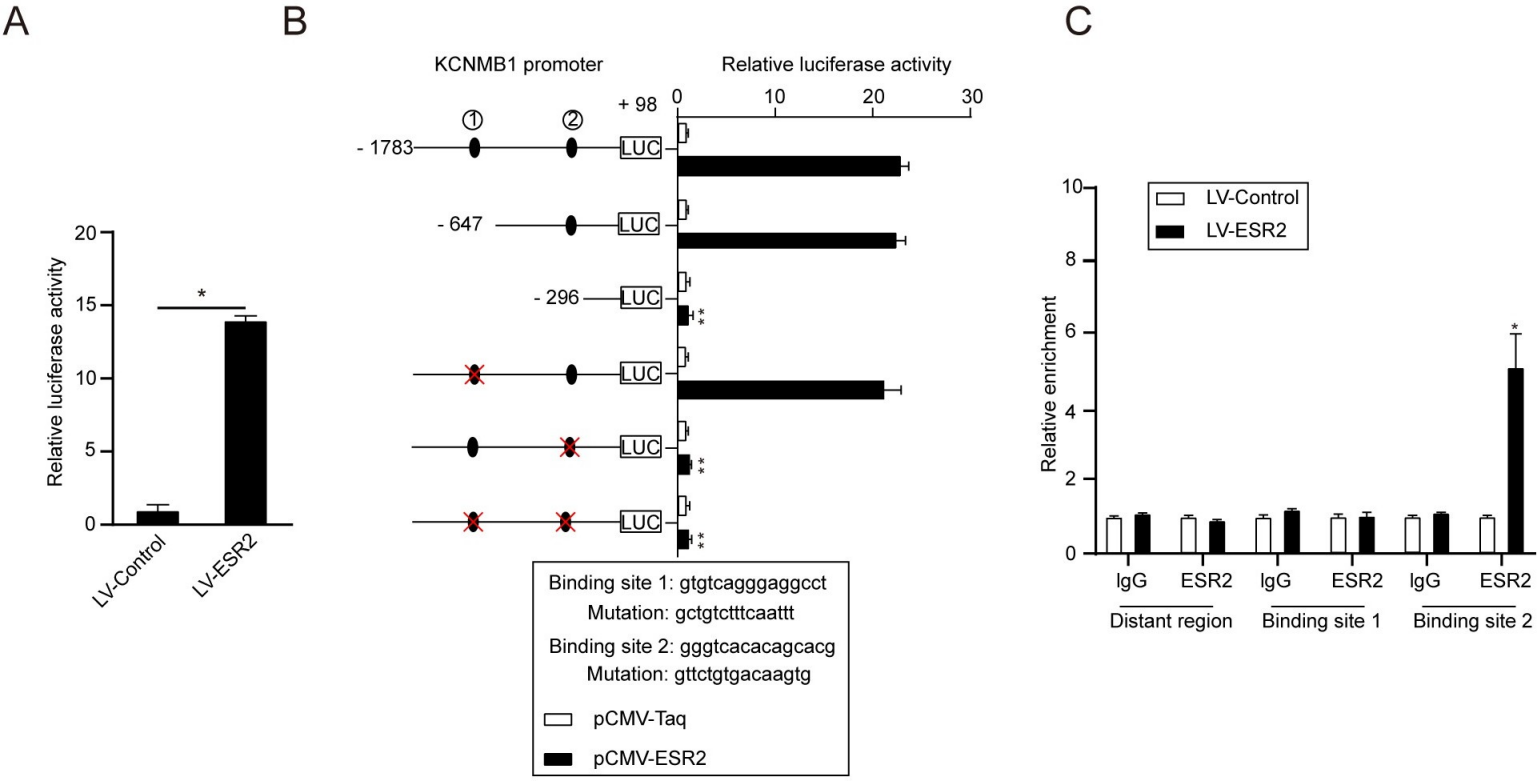
KCNMB1 is a direct transcriptional target of ESR2. **A** Luciferase intensity of the reporter gene driven by the KCNMB1 promoter in HCoSMCs. *P < 0.05 vs. LV-control, n = 3. **B** Luciferase activities of the reporter gene driven by a serially truncated/mutated KCNMB1 promoter with ESR2 binding sites indicated in HCoSMCs. **P < 0.01 vs. KCNMB1 promoter sequence (−1,783/+98), n = 3. **C** ChIP assay for the binding of ESR2 to the KCNMB1 promoter in HCoSMCs. In CHIP and dual luciferase experiments, HCoSMCs were treatment with 50 nmol/l E2.Y-axis shows enrichment with anti-ESR2 antibodies vs. the IgG control. *P < 0.05 vs. anti-IgG, n = 3.

According to queries within the National Center for Biotechnology Information database, KCNMB1 is predicted to be an ESR2 target gene in human colonic SMCs. To evaluate the possibility of ESR2 mediated transcriptional activation of KCNMB1, a dual-luciferase reporter assay was utilized, with cells co-transfected with constructs expressing KCNMB1 and LV-ESR2. In the SMCs transfected with the KCNMB1 promoter, the luciferase intensity was markedly elevated (Fig 6A), thus suggesting that ESR2 does promote KCNMB1 transcriptional activation. Additionally, sequence analysis using JASPAR indicated the presence of two possible ESR2-binding sites within the KCNMB1 promoter. Through serial deletion and site-directed mutagenesis analyses, it was confirmed that ESR2-induced transactivation of KCNMB1 is dependent on binding site 2 (Fig 6B). Moreover, a ChIP assay further verified that ESR2 directly binds to the KCNMB1 promoter (Fig 6C). These results suggest that ESR2 directly binds a specific promoter site on KCNMB1 and subsequently activates gene transcription.

## Discussion

Chronic constipation can seriously affect a patient’s daily life and can cause a significant psychological burden and economic pressure. Furthermore, chronic constipation patients often suffer from gastrointestinal dysmotility [26]. While several studies have shown that E2 reduces gastrointestinal smooth muscle contractions [4, 27, 28], Our in vitro experiments also proved this again. However, a detailed understanding of the mechanisms is still required.

Previous studies have indicated that the regulation of SMC excitability by BKβ1 is accomplished by regulating the colon’s resting potential and action potential activity [14, 22]. Herein, colonic smooth muscle tissue was shown to contract again quickly when IBTX is administered after an Ach-induced maximum contraction. These results suggest that the activation of IBTX-sensitive BKβ1 might inhibit colonic smooth muscle contraction.

BK channels are present in many different tissues, including mammalian smooth muscle tissues [29]. In this study, BKβ1 was found to be widely expressed in rat colonic smooth muscle myometrium. In addition, this finding also indicates that estrogen promotes BKβ1 expression principally by binding to its nuclear receptors and creating a genomic effect. These results are consistent with previous studies where BKβ1 expression levels in the SMCs of uterine arteries responded to E2 treatment via genomic pathways [30].

In a study examining murine colonic myocytes, E2 treatment activated BKβ1 and modulated its excitability [31]. Herein, E2-induced colon relaxation was shown to be partially reversed by IBTX *in vitro*, thus suggesting that BKβ1 may serve as a possible target for estrogenic effects. Moreover, colonic smooth muscle contractions and relaxation have been shown to be closely correlated with intracellular Ca^2+^ concentration [4, 5, 32]. BKβ1 promotes K^+^ outflow by increasing the sensitivity of the pore forming α-subunit to Ca^2+^, which can lead to significant hyperpolarization and smooth muscle relaxation [28, 33]. In this study, E2 was observed to reduce the Ach-induced rise in [Ca^2+^]I, while IBTX reversed the effects. These results suggest that E2 may decrease Ca^2+^ mobilization through a BKβ1 pathway. To further confirm that BKβ1 can affect Ca^2+^ mobilization, secondary validation using shRNA-mediated RNA interference was utilized. These findings suggest that E2 treatment following shBKβ1 transfection can increase intracellular calcium concentrations, with E2 appearing to influence Ca^2+^ mobilization through the β1-subunit.

Previous studies have suggested that in N2A cells, incubation with E2 increases BKβ1 expression in a time- and concentration-dependent manner [33]. In addition, Tang et al. [34] found that E2 can regulate the expression of small-conductance Ca2+ -activated K+ (SK) channels in rat colon smooth muscle cells. In this study, various E2 concentrations and incubation periods were explored to evaluate the relationship between E2 and BKβ1 in rat and human colonic SMCs. Our results were similar to those of Tang et al. [34], but inconsistent with a previous study by Li et al. [35], with this variation possibly attributed to differences in E2 concentrations and the cell types that were utilized. In Fig 2, our experiments illustrated that E2 regulates BKβ1 expression by binding to nERs, it is unclear whether E2 regulates BKβ1 expression in colonic smooth muscle by binding ERα or ERβ. In another study examining GT1-7 cells, a gonadotropin-releasing hormone neuronal cell line, BK currents were shown to be augmented by E2 at physiological concentrations via ERβ [36]. Furthermore, another study also showed that E2 actions on BK subunits were reduced in the presence of siERβ, but not with siERα [35]. Herein, DPN, but not PPT, significantly enhanced BKβ1 expression in human and rat colonic SMCs, thus suggesting that E2 possibly promotes BKβ1 expression via ERβ.

In most cases, estrogens trigger target gene expression by means of ERα or ERβ [37]. However, it is currently unclear whether KCNMB1 is a direct downstream target gene of ESR2. To further explore the potential targeting relationship between ESR2 and KCNMB1, serial deletion, site-directed mutagenesis and ChIP were used in human colonic SMCs. These experiments showed that KCNMB1 is specifically targeted by ESR2, which subsequently results in its transactivation via direct promoter binding.

The increase in Ca^2+^ levels during smooth muscle contraction is due to the release of endoplasmic reticulum stored calcium or the inflow of extracellular Ca^2+^ [38]. In this study, the ERβ/BKβ1 signaling pathway was found to inhibit Ca^2+^ mobilization, but the specific mechanisms modulating this process require further elucidation. Therefore, further electrophysiological experiments are needed to explore the specific mechanisms of Ca^2+^ mobilization and further experimentation is required to confirm our findings. In addition, studies have shown that estrogen can affect gut microbiota [39], and changes in gut microbiota can further affect intestinal motility [40]. Due to time and funding constraints, we did not study the gut microbiota. This is also a direction for our further study.

## Conclusion

In summary, this study revealed the following: (I) E2 inhibits rat colonic smooth muscle contractions and reduces the [Ca^2+^]i concentration by activating BKβ1; (II) E2 increases BKβ1 expression in an ERβ-dependent manner; and (III) KCNMB1 is a direct downstream target gene of ESR2. These findings support the idea that E2 modulates rat colonic smooth muscle relaxation via ERβ/BKβ1 signaling. Overall, these findings could help to improve the current understanding of chronic constipation in women.

## Supporting information

S1 Fig The mean (±SEM) values of the integrated optical density as analyzed by ImageJ software. *P <0.05 versus C, BSA-E2 and EI groups.
(TIF)

S1 Raw images (PDF)
