# Supplementary figures and images for: Estrogen inhibits colonic smooth muscle contractions by regulating BKβ1 signaling
Source: PLoS One. 2023 Nov 10;18(11):e0294249. doi: 10.1371/journal.pone.0294249 (PMC10637685; doi:10.1371/journal.pone.0294249)

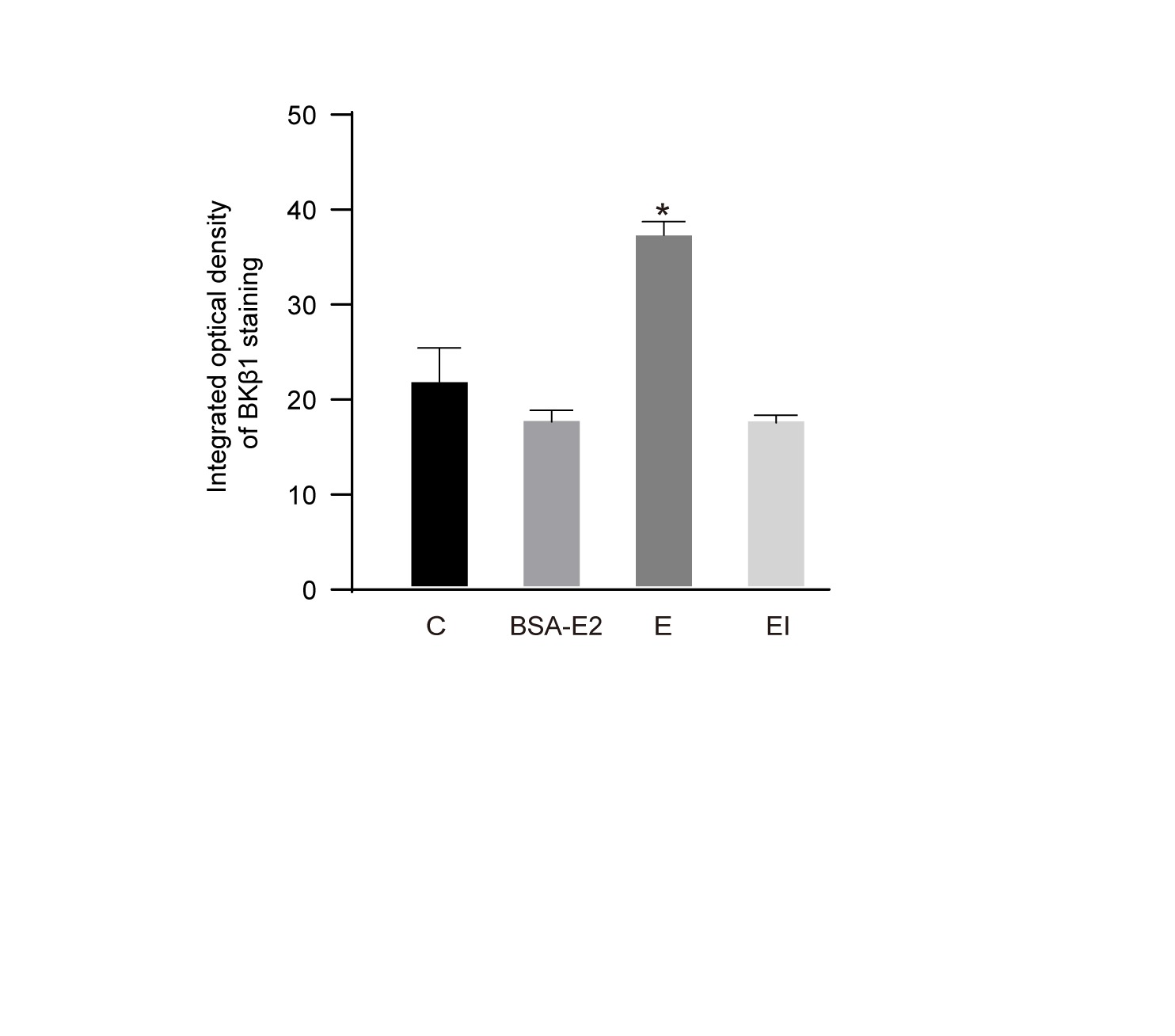

Supplement: S1 Fig — *P <0.05 versus C, BSA-E2 and EI groups. (TIF) [file pone.0294249.s001.tif]
