# Supplementary material for: Estrogen inhibits colonic smooth muscle contractions by regulating BKβ1 signaling
Source: PLoS One. 2023 Nov 10;18(11):e0294249. doi: 10.1371/journal.pone.0294249 (PMC10637685; doi:10.1371/journal.pone.0294249)
Supplement: S1 Raw images — (PDF) [file pone.0294249.s002.pdf]

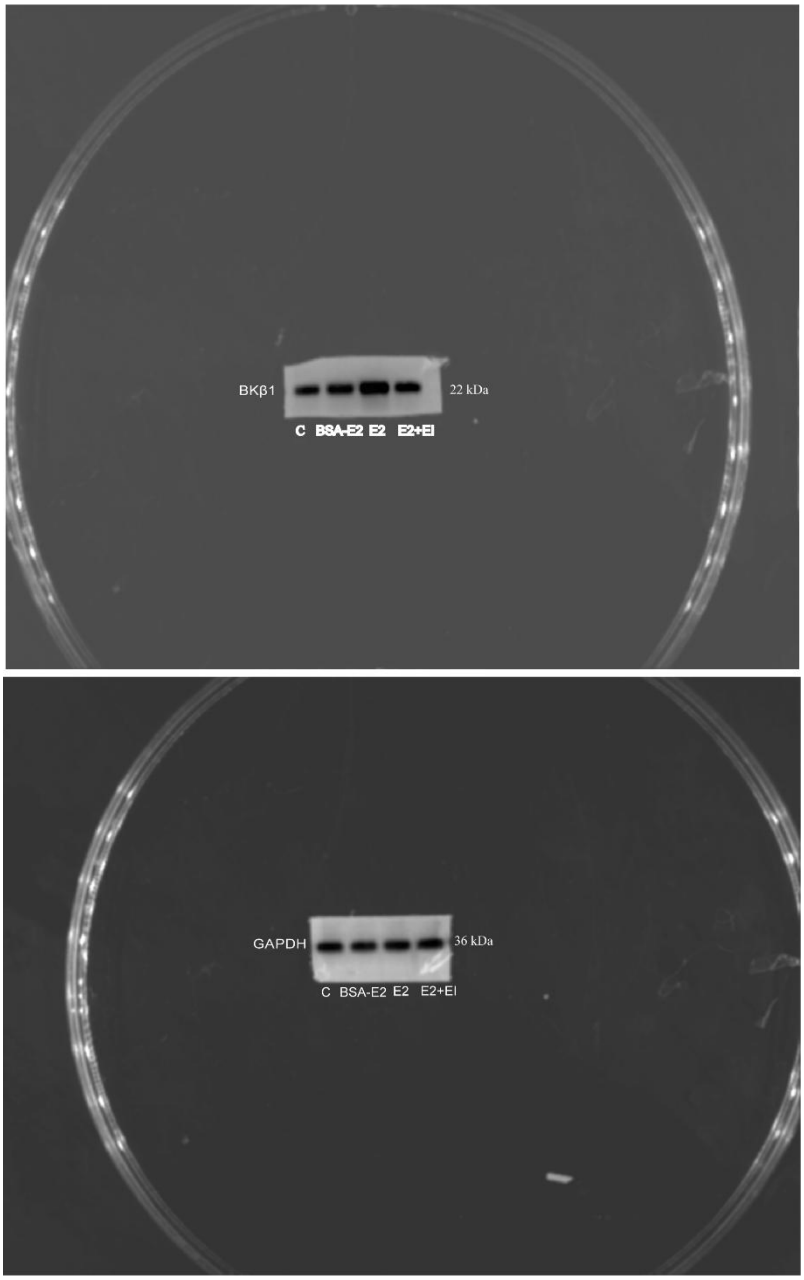

Fig2: BKβ1 expression and distribution in rat colonic smooth muscle

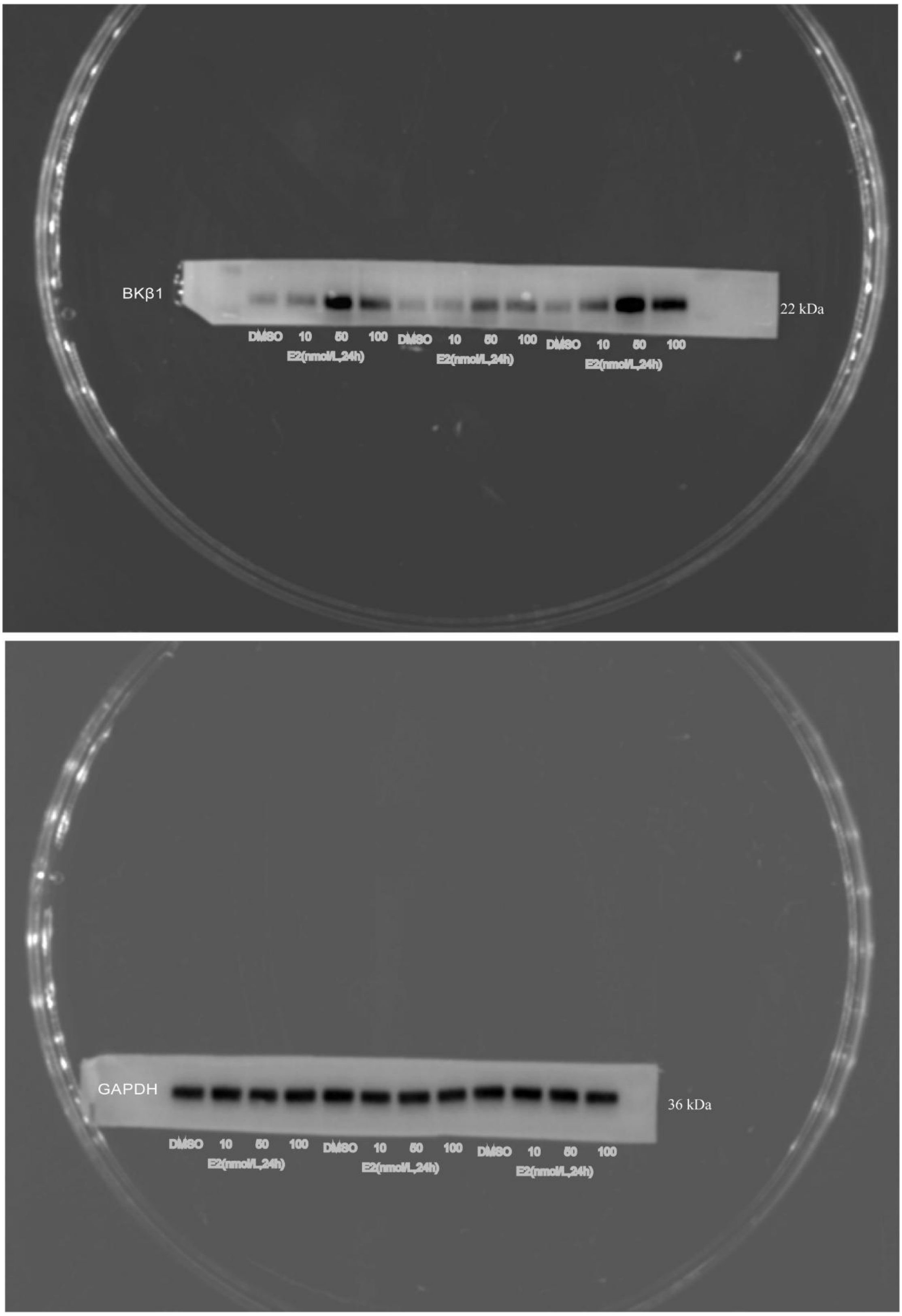

Fig3A. E2 was administered to rat CoSMCs for 24 h at 0, 10, 50, or 100 nmol/l.

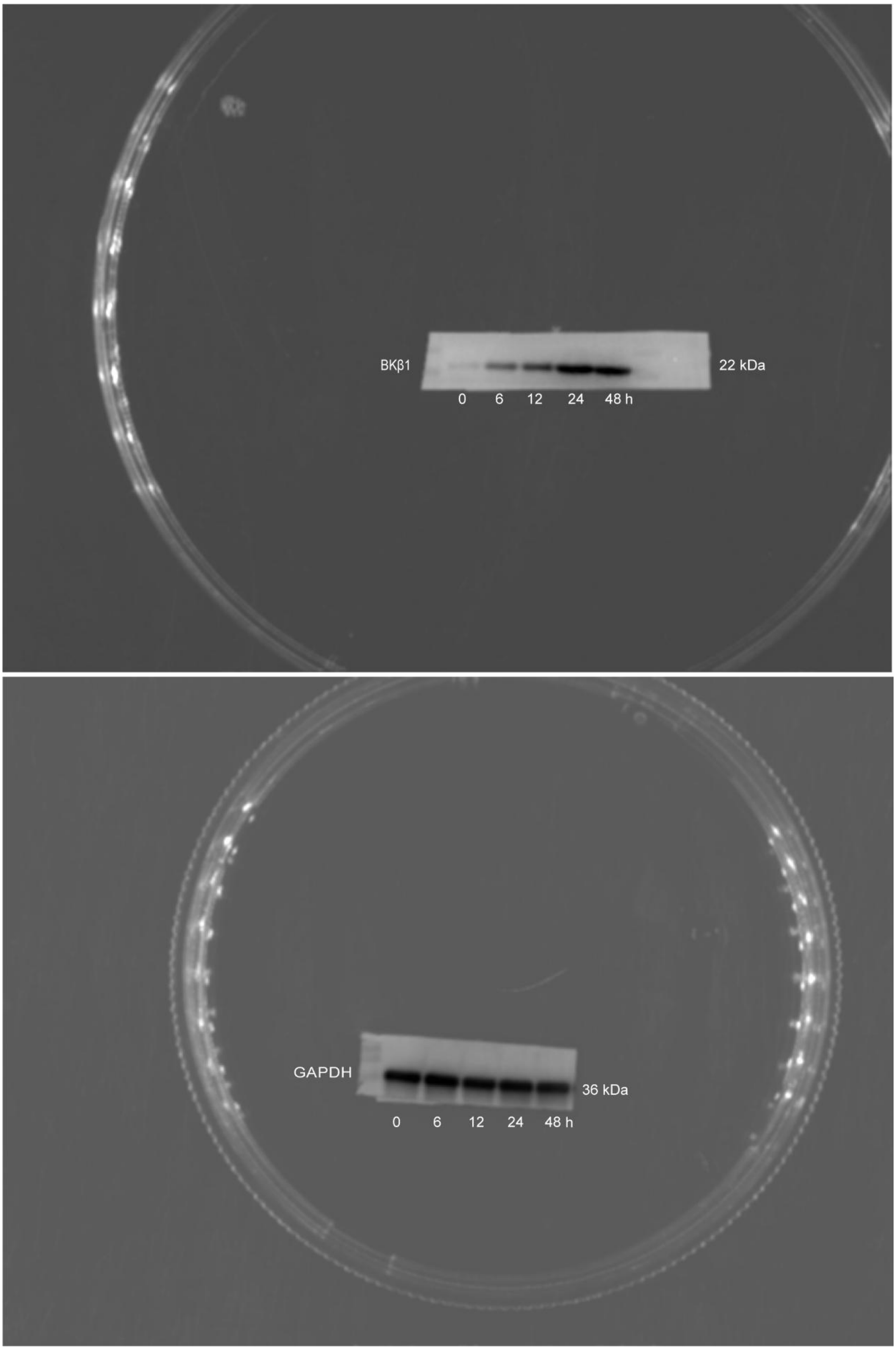

Fig3C. Rat CoSMCs were treated with 50 nmol/l of E2 for 0, 6, 12, 24 or 48 h

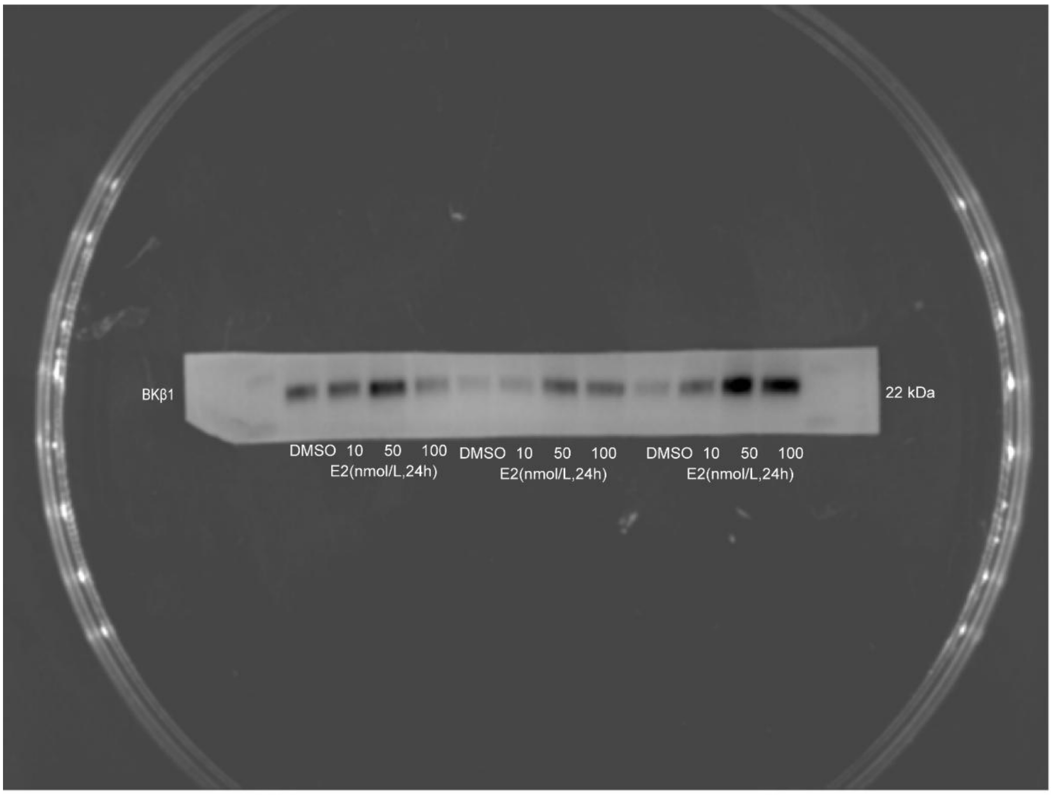

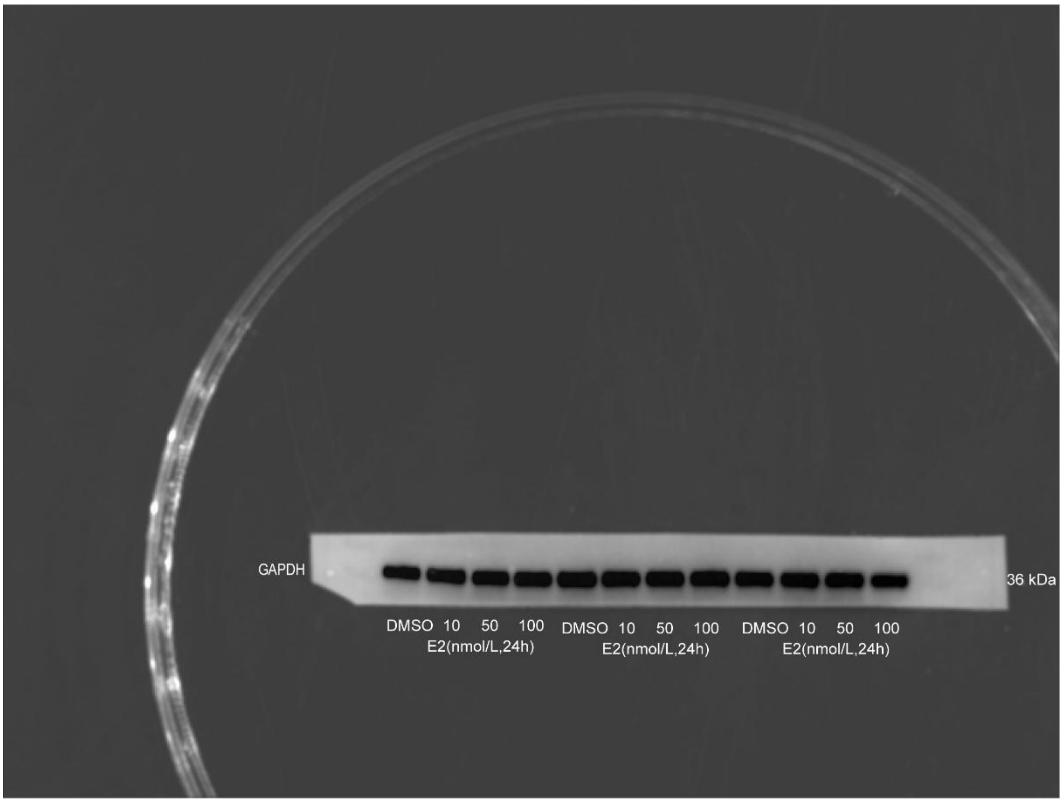

Fig3E:E2 was administered to HCoSMCs for 24 h at 0, 10, 50, or 100 nmol/l

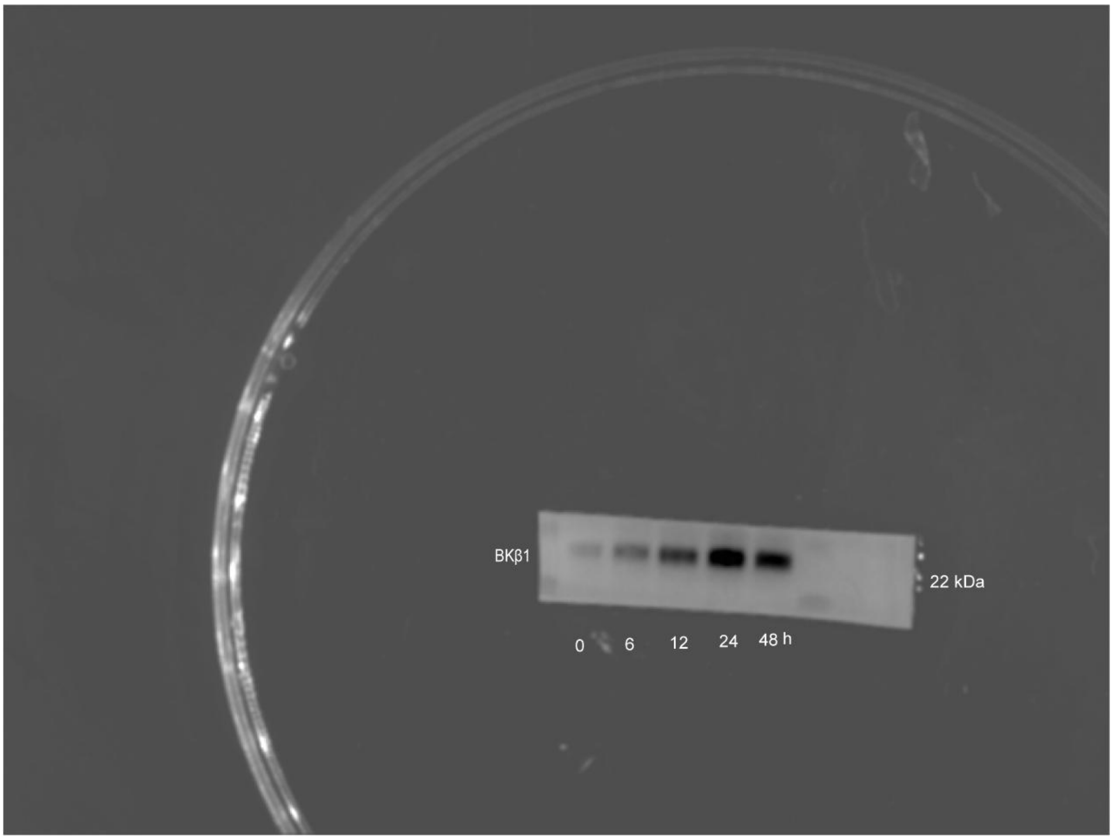

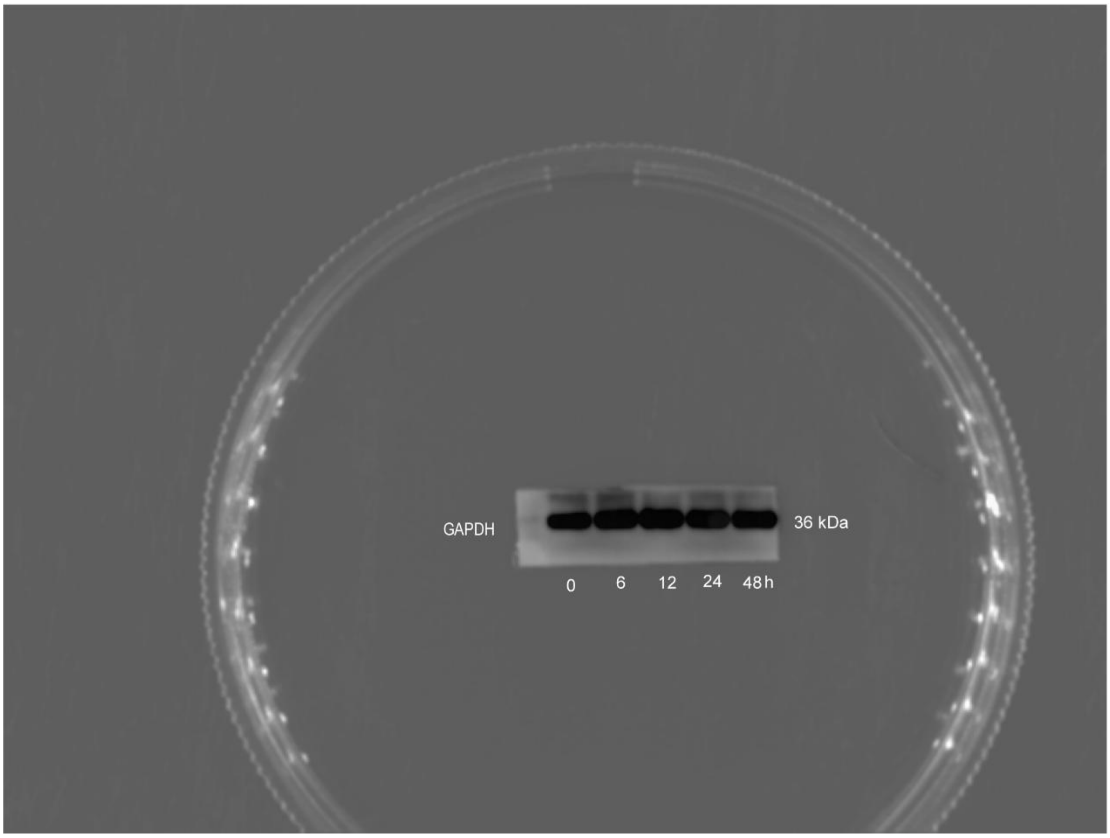

Fig3G: HCoSMCs treated with 50 nmol/l of E2 for 0, 6, 12, 24 or 48 h.

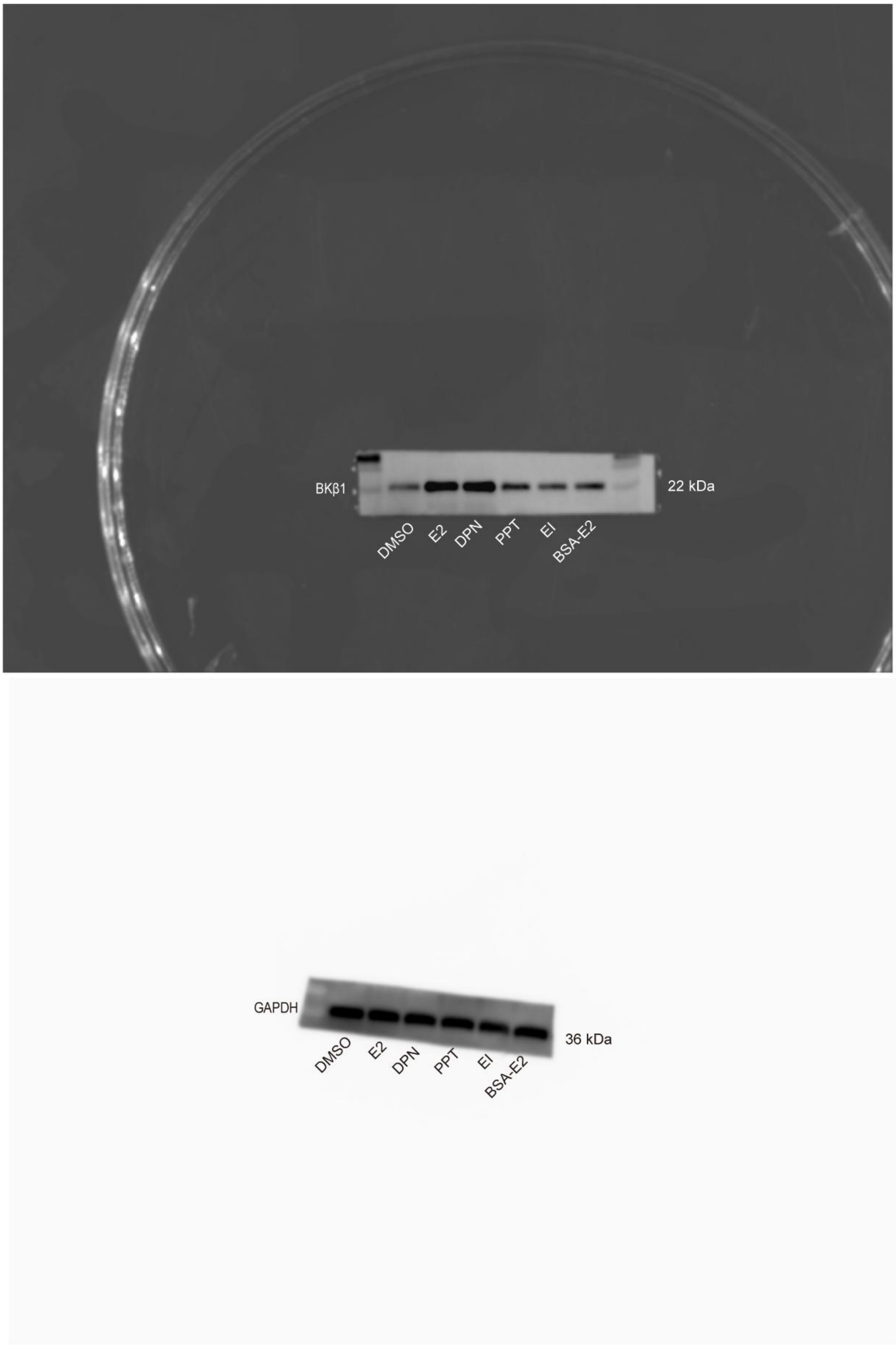

Fig4A:E2 promoted BKβ1 expression in rat CoSMCs in an ERβ-dependent manner

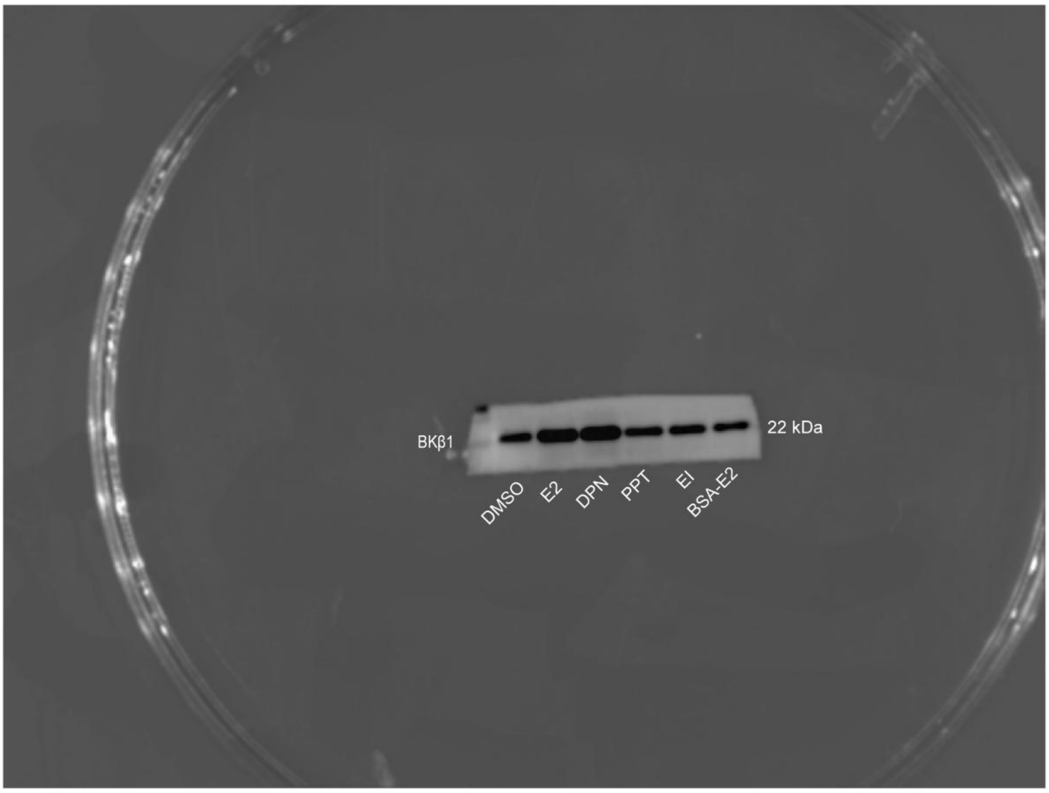

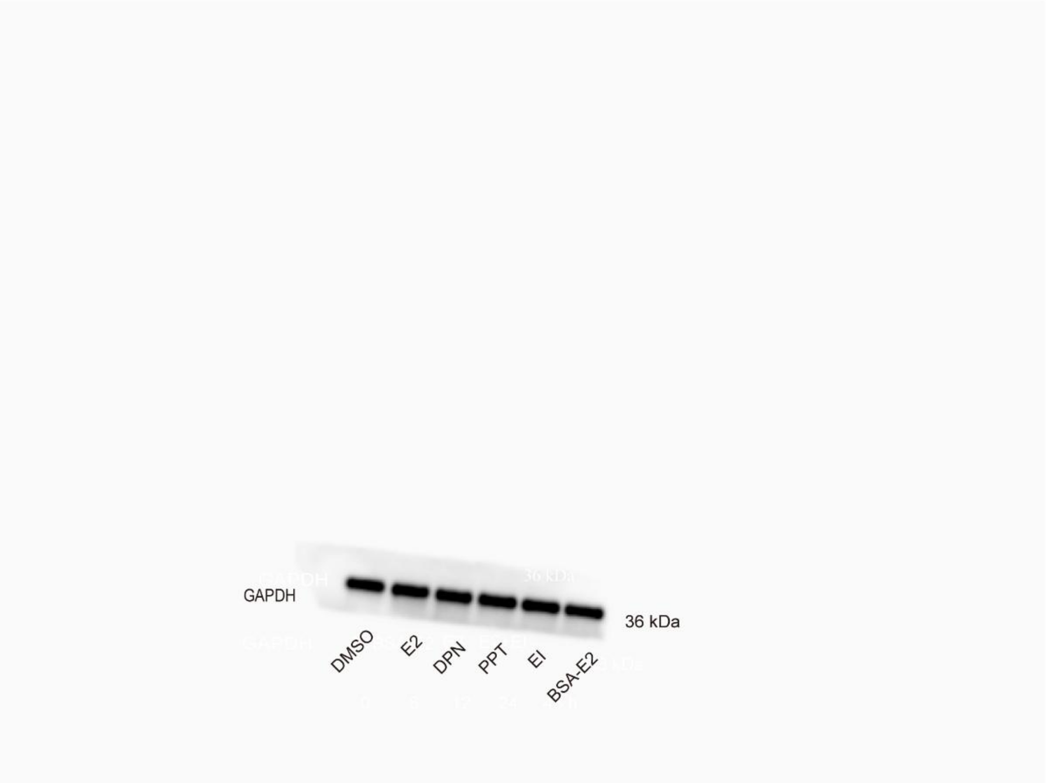

Fig4B:E2 promoted BKβ1 expression in HCoSMCs in an ERβ-dependent manner
